# An investigation of morphological awareness and processing in adults with low literacy

**DOI:** 10.1017/S0142716413000222

**Published:** 2015-03

**Authors:** ELIZABETH L. TIGHE, KATHERINE S. BINDER

**Affiliations:** Florida State University; Mount Holyoke College

## Abstract

Morphological awareness, which is an understanding of how words can be broken down into smaller units of meaning such as roots, prefixes, and suffixes, has emerged as an important contributor to word reading and comprehension skills. The first aim of the current study was to investigate the contribution of morphological awareness independent of phonological awareness and decoding to the reading comprehension abilities of adults with low literacy. Results indicated that morphological awareness was a significant unique predictor of reading comprehension. A second aim of the study was to investigate the processing of morphologically complex words of adults with low literacy in both an oral reading passage and a single-word naming task. Adults’ accuracy and response times were measured on different types of morphologically complex words and compared with control words matched on frequency in both the passage and the naming tasks. Results revealed that adults were vulnerable to morphological complexity: they performed more accurately and faster on matched control words versus morphologically complex word types. The educational implications for Adult Basic Education programs are discussed.

Literacy, the ability to extract meaning from written text, is an invaluable skill enabling individuals to function in daily life. The 2003 National Assessment of Adult Literacy, administered to a nationally representative sample of almost 20,000 adults, reports that approximately 14% of American adults read below the basic literacy level and an additional 22% read at the basic literacy level (Kutner et al., 2007). Research has shown that poor literacy skills are perpetuated through generations. Children of adults with low literacy skills are disadvantaged upon school entrance, which eventuates in a higher probability of dropping out (Kirsch, Jungeblut, Jenkins, & Kolstad, 1993). To most effectively curb this problem, we must understand the process by which adults acquire language and develop reading skills.

Adult Basic Education (ABE) programs are designed to help diminish the problem of adult low literacy by providing adults (ages 16 and older), who are not concurrently enrolled in kindergarten to Grade 12 education, with instruction and coursework to earn a General Educational Development (GED) certificate. These programs serve approximately 2.6 million adults annually; however, this is just a small fraction of the approximately 90 million US adults with low literacy (National Research Council, 2012). Despite the prevalence of low literacy skills among adults, there is a paucity of rigorous research investigating the reading skills and best instructional approaches for this population. Several factors exacerbate the need for high-quality research on adult reading skills and the efficacy of ABE programs: a lack of systematic curriculum and testing materials, high attrition rates, and heterogeneous demographics of the adult population.

The current study enhances the existing body of literature by investigating morphological awareness, a conscious understanding of how words can be broken down into smaller units of meaning, and reading comprehension in adults enrolled in ABE programs (Carlisle, 2000). In order to effectively evaluate the literacy skills for this population, it is important to understand that adults might follow a unique developmental trajectory when acquiring language and reading skills (Perin, 1988; Thompkins & Binder, 2003). Unfortunately, most research has focused exclusively on children's acquisition of literacy skills. Thus, many ABE programs utilize testing materials, instructional methods, and models of reading that were developed for children. Although research on children can help guide literacy research for struggling adult readers, adults and children differ in several important reading areas such as exposure to printed word, experience with language, and ability to use higher order cognitive functioning (Adams, 1990; Hoffman, 1978; Perin, 1988; Thompkins & Binder, 2003).

## READING SKILLS OF ADULTS WITH LOW LITERACY

The limited amount of existing literature on adults with low literacy has suggested that adults across a range of skill levels in ABE programs have deficient decoding, phonological, receptive vocabulary, fluency, rapid automatized naming, and reading comprehension skills (Greenberg, Ehri, & Perin, 1997, 2002; MacArthur, Konold, Glutting, & Alamprese, 2010; Mellard & Fall, 2012; Mellard, Woods, & Fall, 2011; National Research Council, 2012; Sabatini, 2002; Sabatini, Sawaki, Shore, & Scarborough, 2010; Thompkins & Binder, 2003). Greenberg et al. (1997, 2002) reported that ABE students outperformed reading-achievement matched children on word recognition tasks requiring orthographic knowledge (such as sight-word reading). Conversely, children outperformed ABE students on word recognition tasks requiring phonological knowledge (such as nonword reading). Similarly, Thompkins and Binder (2003) reported that adult literacy students had weaker phonological skills and more advanced orthographic knowledge than children matched on reading achievement levels. Comparing ABE students to skilled adult readers, Binder and Borecki (2008) found that adults with low literacy relied more on orthographic information and contextual clues than on phonological information when silently reading connected texts that contained homophone pairs.

To date, no studies with the adult low literacy population have investigated the construct of morphological awareness. Morphological awareness falls under the general umbrella term linguistic awareness, which also encompasses phonological and orthographic awareness. All three linguistic awareness skills are necessary to facilitate reading development and word learning (Berninger, Abbott, Nagy, & Carlisle, 2010). Adult literacy research has found that ABE students have particularly strong orthographic abilities and have consistent deficiencies in decoding and phonological abilities. However, the third component, morphological awareness, has not been investigated in the context of adult literacy. Thus, the current study wants to expand the body of literature by assessing morphological processing and the contribution of morphological awareness, phonological awareness, and decoding to reading comprehension in adults with low literacy.

## MORPHOLOGICAL AND PHONOLOGICAL AWARENESS

Phonological awareness, the ability to distinguish and manipulate the sound structure of language, has consistently been found to be an important predictor of children's reading comprehension (Adams, 1990; Good, Gruba, & Kaminski, 2001). The English orthography does not rely exclusively on the alphabetic principle because grapheme to phoneme correspondences are not always mapped perfectly one to one. Instead, English has a deep orthography, which implies that a single sound (phoneme) can be represented by several graphemes (e.g., *bare* and *bear*). Thus, the English orthography is considered morphophonemic, meaning words are characterized simultaneously by sounds (phonemes) and meanings (morphemes). Phonemes are individual sound units that can build complex words, decompose complex words, or be moved around to form new words. Similarly, morphemes are the smallest units of meaning (e.g., prefixes and suffixes), which can be used to build complex words, decompose complex words, and be moved to form new words. If the English language were purely based on phonetics, words would be spelled the way in which they sound (e.g., the past tense of *trap* would be *trapt*). Since English spelling encompasses both morphological and phonological components, readers understand that *trap* becomes *trapped.*

Several studies have tried to disentangle phonological awareness and morphological awareness and assess the unique contributions of these two constructs to children's reading skills. Jarmulowicz, Hay, Taran, and Ethington (2008) constructed a model to assess the relationship between phonological and morphological awareness and found that they are correlated yet distinct literacy skills. Phonological awareness has a greater impact on reading skills up until third grade, whereas morphological awareness builds on phonological abilities and becomes a more important predictor of reading skills after third grade and through the high school years. Berninger et al. (2010) conducted a longitudinal study investigating growth in phonological, orthographic, and morphological awareness for first through sixth graders. The study reported that phonological and orthographic awareness exhibited the greatest growth during the earlier elementary school years. Growth in morphological awareness was primarily during first through fourth grades; however, growth in derivational morphological awareness showed considerable growth beyond the fourth-grade level. These researchers concluded that phonological awareness is necessary but not sufficient on its own to account for learning to read. Thus, they deduced that orthographic and morphological awareness should be included in reading models. Moreover, morphological awareness displayed the longest developmental trajectory, with growth on different facets of morphological awareness spanning from first through sixth grades. Finally, several studies have investigated whether morphological awareness is predictive of reading skills (both comprehension and word-level reading) after controlling for phonological awareness (Deacon & Kirby, 2004; Kirby et al., 2012; Singson, Mahony, & Mann, 2000). This research highlights the importance of distinguishing between phonological and morphological awareness and their independent contributions to reading comprehension.

## MORPHOLOGICAL AWARENESS AND READING ABILITIES

Several studies have documented that morphological awareness is an important predictor of children's reading comprehension across a range of grades (Carlisle, 1988, 2000; Deacon & Kirby, 2004; Kirby et al., 2012; Nagy, Berninger, & Abbott, 2006; Nagy, Berninger, Abbott, Vaughan, & Vermeulen, 2003). Carlisle (2000) found that morphology tasks accounted for 43% of variance in reading comprehension for third graders and 55% of the variance in reading comprehension for fifth graders. Nagy et al. (2006) investigated the role of morphological awareness, phonological memory, and phonological decoding in predicting reading comprehension, reading vocabulary, and spelling in fourth/fifth graders, sixth/seventh graders, and eighth/ninth graders. For the two younger groups, morphological awareness emerged as the only unique predictor of reading comprehension. For the eighth and ninth graders, all three skills (morphological awareness, phonological memory, and phonological decoding) were unique predictors of reading comprehension; however, morphological awareness still accounted for the greatest percentage of variance.

In addition, morphological awareness has been found to be a strong of predictor of single-word reading with children across a wide range of grades (Deacon & Kirby, 2004; Roman, Kirby, Parrila, Wade-Woolley, & Deacon, 2009; Singson et al., 2000). Singson et al. (2000) found that morphological awareness contributed uniquely to reading abilities (word reading and decoding skills) for third through sixth graders over and above phonological awareness and receptive vocabulary knowledge. Kirby et al. (2012) reported that independent of verbal and nonverbal IQ and phonological awareness, morphological awareness was a significant predictor of word reading accuracy and speed as well as reading comprehension in first through third graders. The current study investigated accuracy and response times on morphologically complex words embedded in a passage as well as morphologically complex words in a single-word recognition task. In addition, because morphological awareness has been found to be a strong predictor of both reading comprehension and single-word decoding, the present study looked at the contribution of morphological awareness over and above the contributions of decoding and phonological awareness to reading comprehension.

## MORPHOLOGY AND CONTEXT

Contextual clues contained in printed texts have been found to promote the processing and understanding of unfamiliar morphologically complex words (Sternberg, 1987). Anglin (1993) reported that context effects begin to play a more important role in word learning after the third grade because children are able to apply morphological analysis to infer the meaning of new words. The average fifth grader reads approximately 1 million words of text in a year and of those words, approximately 15,000 to 55,000 different words are novel to the reader (Nagy, Herman, & Anderson, 1985). Moreover, morphologically complex words account for more than 60% of the vocabulary words children encounter during reading after fourth grade (Egan & Pring, 2004; Nagy & Anderson, 1984; Nagy, Anderson, Schommer, Scott, & Stallman, 1989). Thus, the ability to engage in morphological decomposition and utilize contextual clues in printed text becomes important at increasing grade levels to identifying novel morphologically complex words.

Wysocki and Jenkins (1987) tested fourth, sixth, and eighth graders on their abilities to use morphological and contextual information to define unfamiliar words. The researchers investigated the use of sentence context by presenting unfamiliar words in strong and weak contexts. A strong context was defined by including clues that would help students infer the meaning of the word, whereas a weak context included little or no indication of word meaning. The sixth and eighth graders were better at combining contextual and morphological clues than were the fourth graders, showing significant improvement in identifying unfamiliar word meanings in both strong and weak contexts. The ability of older students to combine information from both morphological rules and context demonstrates a hypothesis set out by Nagy and Anderson (1984) that morphological rules and context work together. Several studies have suggested that adults with low literacy might compensate for poor word decoding skills by developing compensatory strategies such as utilizing contextual clues when reading connected text (Binder & Borecki, 2008; Blalock, 1981; Read & Ruyter, 1985). The current study investigated the ability of adults with low literacy to utilize contextual clues by measuring accuracy on morphologically complex words presented in connected text versus words presented in isolation in a word recognition task.

## DEVELOPMENT OF INFLECTIONAL AND DERIVATIONAL MORPHOLOGICAL RULES

Morphological research differentiates between two types of morphologically complex words: inflected and derived (Carlisle, 2003). Inflectional morphemes alter the tense or pluralize the root word but keep the word class intact (e.g., *pull* to *pulled*). Derivational morphemes can change the meaning of the root word and can also alter the word class (e.g., *help* to *helpless*). Children are able to apply inflectional morphology as early as the preschool years (Berko, 1958; Brittain, 1970; Clark & Hecht, 1982). Although preschoolers have some knowledge of inflectional endings, they have more difficulty with more complex inflectional endings, such as knowing to apply –*es* rather than just –*s* (Berko, 1958). Studies have reported that young children have a tendency to produce overregulation errors when applying the past tense inflection –*ed* (e.g., *runned*; Brown, 1973; Marcus et al., 1992). These types of errors demonstrate that children implicitly understand inflectional morphological rules even though these words are orthographically and phonologically incorrect.

Knowledge and awareness of derivational morphology begins as early as preschool but rapidly increases from first to fourth grades and continues to develop until adulthood (Carlisle, 1988, 2003; Clark, 1982). Preschoolers have the ability to understand simple derivational affixes such as adding the *–er* agentive suffix to words (e.g., *teacher*); however, they have more difficulty applying derivational affixes to words that undergo orthographic and/or phonological shifts (Anglin, 1993). Most studies of morphological awareness have focused exclusively on inflected morphology in younger children or exclusively on derivational morphology in older children; however, in order to understand morphological awareness as a broader construct, it is important to incorporate both inflected and derived morphology within a single study.

Some studies with adult literacy students matched with children on achievement level have reported that adults consistently make morpheme ending errors when spelling both inflected and derived words (Liberman, Rubin, Duques, & Carlisle, 1985; Viise, 1996; Worthy & Viise, 1996). For example, Worthy and Viise (1996) reported that adults made many omissions of inflectional endings—*crack* for *cracking*. Furthermore, the adults made guesses on unfamiliar words that were not related semantically. These findings suggest that adults with low literacy skills have not yet mastered inflected and derived morphologically complex words; therefore, it is imperative to include both word types in morphological tasks for this population.

## MORPHOLOGY AND WORD FREQUENCY AND TRANSPARENCY

Morphological research has also investigated the role of word frequency and familiarity in determining a reader's awareness of the morphological structure of words (Carlisle, 2003; Carlisle & Katz, 2006; Egan & Pring, 2004; Reichle & Perfetti, 2003). Carlisle and Katz (2006) found that the frequency of derived words, root words, and the size of the word family are important in assisting with word recognition. All words have frequency values determined by the Standard Frequency Index (SFI; Carroll, Davies, & Richman, 1971). Words with greater SFIs are more familiar to elementary school children and, thus, more rapidly identified and processed than those with lower SFIs (Larsen & Nippold, 2007).

In addition, the word characteristics of transparency and opaqueness are important in determining a reader's awareness of the morphological structure of words (Carlisle, 2000, 2003; Carlisle & Stone, 2005). Phonological transparency refers to base words that are intact in the derived form of the word: *growth* is phonologically transparent because the root word, *grow*, retains its pronunciation. In contrast, phonological opaqueness refers to a pronunciation change from the base word to the derived word form: *finality* represents a phonological shift because the pronunciation of *final* changes.

Carlisle and Stone (2005) utilized measures of frequency and transparency in their study examining the role of morphemes in the speed and accuracy with which lower and upper elementary students read derived words. In the first part of the study, students were presented with individual words transparent in both spelling and sound. Words contained two morphemes (*hilly*) and single-morpheme, pseudocomplex derived words (*silly*) and were matched for spelling, word length, and word frequency. The students were also presented with low-frequency derived words, all of which contained high-frequency base forms (*puzzlement*), to investigate the role of base word familiarity on speed and accuracy in word recognition. Results indicated that all of the students read derived two-morpheme words more accurately and faster than they did single-morpheme words. In addition, upper and lower elementary students were more accurate and faster at identifying high-frequency derived two-morpheme words as compared with derived low-frequency words with high-frequency bases. However, there was a greater difference in accuracy and speed for high- versus low-frequency words in lower elementary students compared to upper elementary students. Thus, upper elementary students were better able to recognize the high-frequency bases in order to facilitate recognition of low-frequency words.

The second part of the Carlisle and Stone (2005) study investigated the speed and accuracy in which middle and high schoolers read derived words that differed in phonological transparency (shift and stable words). The researchers compiled a list of phonological shift words (e.g., *majority*) and stable words (e.g., *maturity*), which controlled for spelling, word length, base frequency, and derived-word frequency. Previous research indicates that words undergoing phonological shifts present difficulties for children learning to read (Carlisle, 2000). The researchers hypothesized that middle school students would read stable words faster and more accurately than shift words, but they posited that this difference would disappear by high school. However, the results indicated that both middle and high schoolers were more accurate on stable versus shift words.

## CURRENT STUDY

The present study had two primary aims. First, we investigated the contribution of morphological awareness independent of phonological awareness and decoding to reading comprehension in adults enrolled in ABE programs. Within an adult literacy framework, past research has investigated the linguistic awareness constructs of phonological and orthographic knowledge. We wanted to expand the existing research by looking at the third component of linguistic awareness: morphological awareness. Previous research with children has found that morphological awareness is an important predictor of reading comprehension after controlling for phonological awareness (Deacon & Kirby, 2004; Kirby et al., 2012). We wanted to assess if morphological awareness was also a unique contributor to reading comprehension for adults with low literacy.

Second, the other aim of the current study was to examine morphological processing in adults with low literacy by measuring accuracy and response times on different types of printed morphologically complex words presented in an oral reading passage and a single-word naming task. If morphological awareness was found to be an important contributor to reading comprehension for this population, we wanted to assess what aspects of morphological complexity adults were sensitive to. In accordance with a broader definition of morphological awareness, we included both inflected and derived word types. In addition, the study incorporated derived words that were phonologically transparent, opaque, and included high- and low-frequency words from the Carlisle and Stone (2005) study. By including a passage and a single-word recognition task, we were able to look at the role of context in the accuracy and response times of identifying morphologically complex words.

The present study addressed four research questions:

1. Does morphological awareness account for additional variance, independent of phonological awareness and decoding skills, for adults with low literacy skills?
2. How accurate and how fast are response times of adults with low literacy skills on inflected morphologically complex words compared with single-morpheme matched control words in an oral reading passage and a single-word naming task?
3. How accurate and how fast are response times on derived morphologically complex words compared with matched control words in an oral reading passage and a single-word naming task?
4. Does context influence accuracy?

We hypothesized that similar to research with children, morphological awareness would make a unique contribution to reading comprehension independent of phonological awareness and decoding skills for adults with low literacy (Deacon & Kirby, 2004; Kirby et al., 2012). We also hypothesized that adults would be more accurate and faster at responding to matched control words as compared with morphologically complex words on all word types. A difference in accuracy and response times between morphologically complex words and matched control words would indicate sensitivity to morphological complexity. Similar to Carlisle and Stone (2005), we predicted that adults would be less accurate and show slower response times on phonological shift compared to stable words and low-frequency words compared to high-frequency words. Based on spelling research with adult literacy students, we hypothesized lower accuracy on inflected endings compared to single-morpheme matched control words (Viise, 1996; Worthy & Viise, 1996).

Previous research has found that children rely on contextual clues and morphological awareness to decipher novel words (Nagy & Anderson, 1984). Further, adult literacy research has shown that adults rely on contextual clues during reading to mask word-decoding difficulties (Binder & Borecki 2008; Read & Ruyter, 1985). Thus, we hypothesized that context would aid ABE students in recognizing unfamiliar morphologically complex words.

## METHOD

### Participants

Participants included 57 adults from an urban ABE program located in western Massachusetts. In this program, adult learners spend 3 hr a day, 5 days a week in the classroom. The majority of this time is spent with a classroom instructor. Most of the instructional time is devoted to teaching the whole group, but some time is spent in breakout groups in which a tutor might work with the group or individuals to develop their skills. This site also has access to a computer lab, so if software is available, the students spend their time on software that is relevant to their lessons. The participants consisted of 39% males (*n* = 22) and 61% females (*n* = 35). Their ages ranged from 18 to 57 years (*M* = 26.9). The racial and ethnic background of the participants was varied: 42% had a Hispanic background, 37% were African American, 18% were Caucasian, and 3% were Asian. There were a few participants (14%) who reported that they had been previously diagnosed with a learning disability. The participants included 12 from the English for speakers of other languages (ESOL) Level 3 class, 13 from the pre-GED (equivalent of 5th- to 8th-grade levels) class, and 32 from the GED (9th- to 12th-grade level). All of the pre-GED adults included in the study were reading at the higher end of the grade level based on their Test of Adult Basic Education—Reading scores. We collected data on their phonological decoding skills and their passage comprehension abilities, and the mean grade level equivalency for these skills were 5.8 (*SD* = 3.31) and 4.4 (*SD* = 2.11), respectively. In addition, all ESOL participants were native Spanish speakers and all were literate in their native language.^1^ The educational background of the participants was varied: 7.7% had fewer than 6 years of formal education, 23% had received some middle school exposure, and 69.2% had spent some time in high school. Our participant demographics are consistent with those reported from ABE programs across the United States and thus considered to be a representative sample (see National Research Council, 2012). Of the 57 participants, only 52 completed both days of testing. Participants received $15.00 as compensation for their time.

### Materials

Participants were administered a battery of literacy assessments measuring morphological, phonological, decoding, and reading comprehension skills. In addition, an experimenter-designed oral reading passage and a single-word naming task were administered.

### Morphological awareness

We incorporated the three tests described below to assess morphological awareness. These morphological awareness tasks were chosen because they were consistently found throughout the morphology literature and consisted of both real-word and nonword tasks. Because these three measures were highly correlated, we created a composite score, referred to as morphological awareness for our analyses. The Cronbach α coefficient for the measures was 0.82.

### Base form morphology task

The base form morphology task, adapted from Leong (2000) and Carlisle (1988), aimed to assess morphological structure by recognition of the root word of derived target words. The participant deconstructed derivational target words into base words. The examiner read aloud a derived target word, which served as a prime for the participant. Next, the examiner read a short sentence with a blank at the end. The participant was expected to provide the base word of the target word given in the beginning of the sentence. For example, “Growth. She wanted her plant to _____”; “grow”. The task included four different conditions: no-change (*growth–grow*), orthographic-change (*foggy–fog*), phonological-change (*popularity*–*popular*), and a both-change (*width–wide*). The task was adapted from previous studies to include equal numbers of no change, orthographic shift, phonological shift, and both shift items. A correct answer received 1 point and an incorrect or no answer received 0 points. Participants were given a practice round of 2 items and then completed seven sentences in each condition for a total of 30 items. The test was discontinued if the participant made six errors. The Cronbach α coefficient for the base form morphology task was 0.97 for the sample.

### Derived form morphology task

This task, adapted from Leong (2000) and Carlisle (1988), was similar in layout to the base form morphology task. Again, the task was adapted to have equal numbers of no change, orthographic shift, phonological shift, and both shift items. The task assessed an individual's ability to transform a base word into a derived word. The examiner provided participants with a base word, followed by a sentence concluding with a blank. The participant was asked to fill in the blank with the proper derived form of the word. For example, “Explain. His excuse was a bad _______”; “explanation.” The same four conditions were used: no change, orthographic change, phonological change, and both change. Correct answers received one point and incorrect or no answers received zero points. A practice round of 2 items was included and participants completed seven sentences in each condition for a total of 30 items. The test ended if the participant made six errors. The Cronbach α reliability coefficient for the derived form morphology task was 0.97 for the sample.

### Derivational Suffix Choice Test of Pseudowords

This test was designed to assess an individual's ability to manipulate morphemes using nonwords (Mahony, 1994; Singson et al., 2000). The test was given to the participants in written form and administered orally to avoid reading difficulties. The test displayed a sentence with a blank, and the participant was prompted to select the appropriate answer from four listed choices. For example, “Our teacher taught us how to _______ long words. Answer choices included *jittling*, *jittles*, *jittled*, and *jittle*. The correct response, *jittle*, received one point, while an incorrect answer or no answer resulted in zero points. Participants were given 14 of these items; however, the test was stopped if the participant made six errors. The Cronbach α reliability coefficient for the Derivational Suffix Choice Test of Pseudowords was 0.84 for the sample.

### Phonological awareness

#### Dynamic indicators of basic early literacy skills phoneme segmentation fluency

The phoneme segmentation fluency subtest measures phonological awareness by testing the ability to break real words into their subsequent phonemes. The examiner presented a word orally to the participant and asked the participant to say all the sounds in the word. For example, if given the word *mop*, the correct response would be “/m/ /o/ /p/.” Participants were timed for 1 min. Participants were required to say each individual sound to receive full credit. The correct number of phonemes per minute determined the phoneme segmentation fluency rate (Good & Kaminski, 2002). Binder, Snyder, Ardoin, and Morris (2011) used this test in an ABE setting and obtained a Cronbach α of 0.87.

### Decoding

We utilized two measures of decoding, which are described below. These two measures were highly correlated and thus we utilized a composite score referred to as decode in our analyses. The Cronbach α coefficient for the two measures was 0.79.

#### Woodcock Reading Mastery Tests—Revised (WRMT-R) word attack and letter word ID

The word attack subtest of the WRMT-R assesses an individual's ability to decode nonwords (Woodcock, 1987). Each participant was presented with 45 nonwords, such as *nat* or *ib*, and asked to read them aloud. A correct response elicited a point only if the whole word was pronounced correctly. No response, incorrect syllable pronunciation, or reading the syllables disjointedly resulted in no points. The test was discontinued if the participant answered 6 incorrectly. This test has a reliability of 0.92 (Woodcock, 1987). Binder et al. (2011) used this test in an ABE setting and obtained a Cronbach α of 0.94.

The letter word identification subtest of the WRMT-R assesses participant's abilities to recognize and pronounce individual letters and words. Participants were shown a binder containing pages with letters and groups of single words and asked to identify specific words/letters. The words increased in difficulty as the task progressed. The test was suspended if the participant answered six words incorrectly. This test has a reliability of 0.97 (Woodcock, 1987). Binder et al. (2011) reported a Cronbach α of 0.95 with ABE students.

### Reading comprehension

The passage comprehension subtest of the WRMT-R is designed to measure readers’ abilities to understand words and sentences by asking participants to rely on pictures and contextual cues (Woodcock, 1987). Participants were presented with a series of pictures with written words and asked to pick the picture that was described by the written words. Next, the participant was presented with sentences, each with a missing word, which participants were asked to supply. Either a picture or contextual cues within the sentence were provided to enable the participant to fill in the missing word. For example, “The drums were pounding in the distance. We could ____ them.” Participants were expected to supply the correct answer, “hear,” and testing was suspended if participants provided six incorrect responses. This test has a reliability of 0.93 (Woodcock, 1987). Binder et al. (2011) reported a Cronbach α of 0.92 with the ABE students.

### Oral reading passage

A narrative passage containing 327 words was constructed with particular attention to different inflectional endings and types of derived words. For inflected endings, the passage included eight –*s* words, eight *–ed* words, and six *–ing* words, each of which was matched based on frequency with single-morpheme control words utilizing the SFI (e.g., *times* and *since*; *walked* and *stay*; *smiling* and *marry*; Appendix A). The passage also included 34 derived words from the Carlisle and Stone (2005) study: low-frequency derived words with high-frequency bases matched with high-frequency, high-frequency base words (e.g., *flowery* and *lucky*); stable versus phonological shift words matched on frequency and word length (e.g., *cultural* and *natural*); and high-frequency, two-morpheme words matched with pseudocomplex derived, single-morpheme words (e.g., *hilly* and *silly*). The derived words from the Carlisle and Stone (2005) study were matched based on spelling, word length, base frequency, and derived-word frequency (Appendix A). The target words were not predicted from the semantic context of the passage. Thus, for the most part, the target words were in a neutral context, but certainly the syntax of the passage aided word identification. This was probably most important for the derived words because the affixes often alter the part of speech.

Participants were asked to read the entire passage aloud while the researcher tape-recorded and marked errors on a score sheet. The same passage was administered to all participants. Number of errors were recorded on a score sheet—“1” for correct and “0” for incorrect. A single scorer recorded all errors during test administration. A random 20% of the passage errors were rescored utilizing the tape-recording to ensure that scoring was accurate. Accuracy was 100% between scoring checks.

### Naming task

The experimenter-designed naming task utilized the same word types as those used in the oral reading passage. All words were matched based on frequency with the corresponding word types in the oral reading passage. For inflected endings, the naming task included three –*s* words, three *–ed* words, and three *–ing* words, each of which was matched based on frequency with single-morpheme control words utilizing the SFI (Appendix B). For derived words, the naming task included 30 words from the Carlisle and Stone (2005) study including: low-frequency derived words with high-frequency bases matched with high-frequency, high-frequency base words (e.g., *beastly* and *icy*); stable versus phonological shift words matched on frequency and word length (e.g., *precision* and *confusion*); and high-frequency, two-morpheme words matched with pseudocomplex derived, single-morpheme words (e.g., *windy* and *candy*). The derived words from the Carlisle and Stone (2005) study were matched based on spelling, word length, base frequency, and derived-word frequency (Appendix B).

Participants were shown a total of 48 words on a computer screen, each of which was presented in isolation for up to 5 s. Participants read each word aloud and the E-Prime computer program recorded the response time data while the researcher kept track of errors. A random 20% of the naming task was rescored by listening to the tape-recording and accuracy was 100%.

### Procedure

The tasks were administered individually to the participants in two 30-min sessions over a 3-day span. The order of the sessions and the order of the tasks within the sessions were counterbalanced. Participants were recruited on a voluntary basis, and the only requirement for participation was a signed informed consent form. Testing took place in a quiet classroom at the center.

## RESULTS

### Regression of morphological awareness, phonological awareness, and decoding on reading comprehension

Table 1 presents the correlations between all morphological awareness, phonological awareness, decoding, and reading comprehension measures. All of these literacy assessments were positively correlated.

**Table 1.** Correlations between literacy assessments

Tasks	1	2	3	4	5	6	7	8	9
1. DMORPH	—	.68**	.74**	.92**	.47**	.58**	.75**	.73**	.77**
2. BMORPH	—	—	.66**	.90**	.45**	.46**	.60**	.59**	.71**
3. Suffix choice	—	—	—	.84**	.39**	.63**	.66**	.71**	.69**
4. MA total	—	—	—	—	.49**	.60**	.74**	.74**	.81**
5. DIBELS PSF	—	—	—	—	—	.54**	.49**	.55**	.57**
6. Word attack	—	—	—	—	—	—	.68**	.89**	.51**
7. Letter word	—	—	—	—	—	—	—	.94**	.60**
8. Decode total	—	—	—	—	—	—	—	—	.61**
9. Passage comp	—	—	—	—	—	—	—	—	—

*Note:* DMORPH, derived form morphology task; BMORPH, base form morphology task; MA, morphological awareness; DIBELS PSF, dynamic indicators of basic early literacy skills phoneme segmentation fluency subtest. *N* = 55 for DMORPH, word attack, DIBELS, letter word ID, and decode total; *N* = 54 for BMORPH and suffix choice; *N* = 52 for MA.

**p < .01.

We performed a multiple regression with the composite decoding score, composite morphological awareness score, and phonological awareness as predictor variables and passage comprehension as the outcome variable. We expected that morphological awareness, phonological awareness, and decoding would be significant predictors of reading abilities for adults with low literacy skills. The regression equation was significant, *F* (3, 48) = 37.70, *p* < .001, accounting for 70.2% of the variance. Both morphological and phonological awareness were significant unique predictors, but decoding was not a unique predictor of reading comprehension (see Table 2).

**Table 2.** Regression analysis of phonological and morphological awareness on reading comprehension tasks

Tasks	β	*SE*	*t*
Phonological awareness	0.25*	0.048	2.66
Morphological awareness	0.75***	0.038	6.30
Decode	−0.08	0.050	−0.67

*Note: N* = 52. *R*^2^ = .702.

*p < .05. ***p < .001.

We also wanted to determine how much additional variance morphological awareness would contribute to the explanation of reading comprehension above phonological awareness. We performed a hierarchical regression with passage comprehension as the outcome measure. In the first block, we entered only phonological awareness, and the regression was significant, *F* (1, 50) = 24.24, *p* < .001, accounting for 32.6% of the variance. In the second block, we included morphological awareness, to see if this addition significantly altered *R*^2^. The overall regression was significant, *F* (2, 49) = 56.97, *p* < .001. Furthermore, morphological awareness explained 37.3% of the variance beyond phonological awareness.

### Accuracy on inflected morphologically complex words

One of our primary questions addressed the word identification accuracy on inflected morphologically complex words as compared with single-morpheme matched control words between words in context (oral reading passage) and isolated words (naming task). We performed a 2 Morphological Complexity (morphologically complex vs. single-morpheme matched control words) × 2 Task Type (oral reading passage vs. naming task) repeated measures analysis of variance (ANOVA) to investigate accuracy levels within participants. Percent variables were calculated to adjust for different total numbers of inflected and matched control words between the passage and naming tasks. A Bonferroni corrected α level of 0.025 was utilized to determine significant effects.

We found a significant main effect of morphological complexity, such that participants were significantly more accurate at reading the matched control words (*M =* 95.6%) as compared with the inflected complex words (*M* = 85.7%), *F* (1, 51) = 45.55, *p* < .001. As expected, this finding demonstrates that adults with low literacy skills are vulnerable to morphologically complex words in print, and they experience difficulty with these words. There was no significant main effect of task type, *F* (1, 51) = 2.52, *p* > .05. Participants performed similarly on inflected words and matched control words in both the oral reading passage (*M* = 91.4%) and the naming task (*M* = 89.9%).

**Figure 1.**
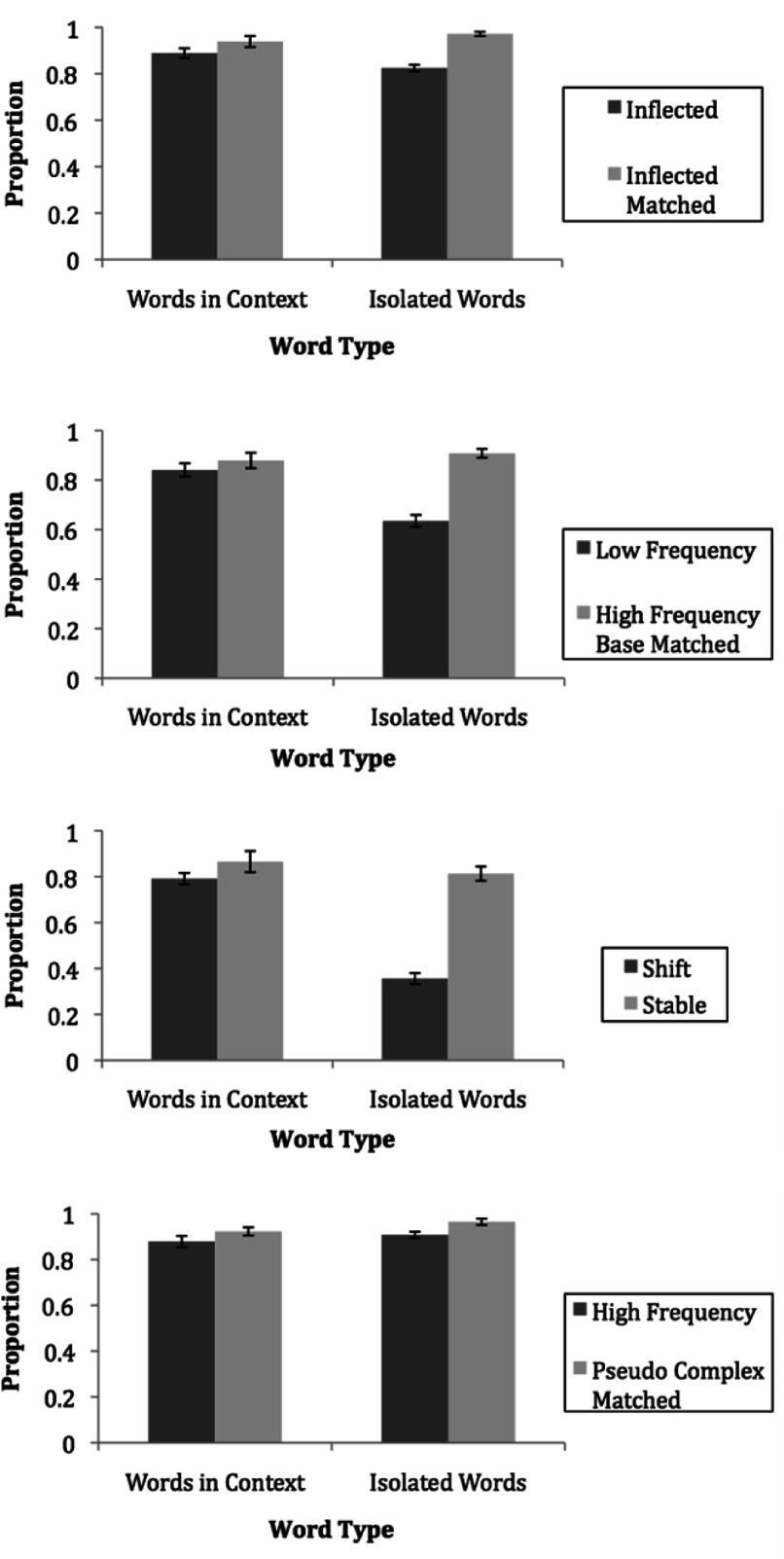
The mean accuracy on all word types in context and in isolation.

There was a significant two-way interaction between morphological complexity and task type, *F* (1, 51) = 19.48, *p* < .001. Within both the oral reading passage and the naming task, single-morpheme matched control words (*M*s = 93.9% and 97.2%, respectively) were read more accurately than morphologically complex words (*M*s = 88.9% and 82.5%, respectively; *p =* .002, *p* < .001). Interaction contrasts revealed that the magnitude of difference in accuracy between inflected complex words and single-morpheme matched controls was significantly larger in isolation as compared with the magnitude of difference in accuracy between inflected complex and single-morpheme matched control words in context, *F* (1, 51) = 19.48, *p <* .001 (see Figure 1, panel 1). These findings demonstrate sensitivity to morphological complexity in printed words, such that ABE students performed more accurately on single-morpheme matched control words versus inflected complex words. Further, ABE students were better at identifying words presented in context as compared to words presented in isolation.

### Accuracy on derived morphologically complex words

We were also interested in printed word identification accuracy on types of derived morphologically complex words versus matched control words presented in context and in isolation. We performed a separate 2 Morphological Complexity (morphologically complex vs. matched control words) × 2 Task Type (oral reading passage vs. naming task) repeated measures ANOVA for each of our three derived word types. Percent variables were calculated to adjust for different total numbers of types of derived words between the oral reading passage and the naming task. A Bonferroni corrected α level of 0.025 was utilized to determine significant effects.

#### Low-frequency, high-frequency base versus high-frequency, high-frequency base words

We found a significant main effect of morphological complexity, such that the high-frequency base matched control words (*M* = 89.3%) were read more accurately than the low-frequency, high-frequency base words (*M* = 73.8%), *F* (1, 51) = 52.12, *p* < .001. As predicted, there was a significant main effect of task type such that participants were more accurate at reading words in context (*M* = 86%) than at reading them in isolation (*M* = 77.1%), *F* (1, 51) = 17.76, *p <* .001. This indicates that context aided the adults with low literacy skills in identifying morphologically complex words.

There was a significant interaction between morphological complexity and task type for low-frequency versus high-frequency base matched control words, *F* (1, 51) = 37.76, *p* < .001. Within the naming task, participants were significantly more accurate on the matched control, high-frequency words (*M* = 90.8%) as compared with the low-frequency words (*M* = 63.5%); however, in context there was no significant difference in accuracy between low-frequency (*M* = 84.1%) and high-frequency base matched control words (*M* = 87.9%; *p <* .001, *p* = .090, respectively; see Figure 1, panel 2). This finding once again demonstrates that adults with low literacy skills are vulnerable to morphological complexity in print and that the adults are more accurate at recognizing words in context than in isolation.

#### Phonological shift versus stable words

We found a significant main effect of morphological complexity, such that stable matched control words (*M* = 83.9%) were read more accurately than phonological shift words (*M* = 57.3%), *F* (1, 51) = 103.32, *p <* .001. There was also a significant main effect of task type, such that printed words were read more accurately in context (*M* = 82.8%) than words in isolation (*M* = 58.4%), *F* (1, 51) = 62.95, *p* < .001.

There was a significant interaction between morphological complexity and task type for phonological shift versus stable words, *F* (1, 51) = 75.36, *p* < .001. Within each task type participants were more accurate at identifying stable words (*M* = 86.5% passage; *M* = 81.3% naming) than shift words (*M* = 79.1% passage; *M =* 35.6% naming; *p =* .001, *p* < .001, respectively). Interaction contrasts demonstrated that the magnitude of difference in accuracy between the shift words and the stable matched control words in isolation was significantly larger than the magnitude of difference in accuracy between the shift and stable words in context, *F* (1, 51) = 75.36, *p* < .001 (see Figure 1, panel 3). These results indicate that adults performed more accurately on the stable matched control words as compared with the shift words and were more accurate reading words in context versus in isolation.

#### Complex versus pseudocomplex words

We found a significant main effect of morphological complexity such that participants were significantly more accurate at reading the pseudocomplex matched control words (*M* = 94.4%) than the morphologically complex words (*M =* 89.3%), *F* (1, 51) = 15.65, *p* < .001. This indicates that adults with low literacy skills are vulnerable to derived morphologically complex words, responding more accurately to pseudocomplex matched control words. There was not a significant main effect of task type, indicating that participants performed similarly reading words in isolation (*M* = 93.7%) and when reading words in context (*M* = 90.1%), *F* (1, 51) = 4.43, *p* < .05. There was also no significant interaction between task type and morphological complexity for morphologically complex versus pseudocomplex matched control words, *F* (1, 51) = 0.21, *p* > .05 (see Figure 1, panel, 4).

### Response times on the oral reading passage and naming task

Response time data were collected for both the oral reading passage and the naming task; however, the way in which response time data were collected differed between the two tasks. For the naming task, the response time was measured from the presentation of the word to the time the participant made his first articulation of the word. For the oral reading passage, the response time was the length of time needed to read the entire word. These data were extracted from the sound files using PRATT software.

Participants’ times for each of the word types were averaged, and any words read incorrectly were eliminated. For the naming task only, data points related to equipment malfunctions were removed: words under 200 ms or above 3000 ms. This resulted in the removal of 3.86% of the words. Finally, for both tasks we purged outliers above 2 *SD* from the mean: an additional 4.69% of words in the naming task and 3.32% of words in the passage.

Although the two measures of response times were different, we ran a 2 Morphological Complexity × 2 Task Type repeated measures ANOVA for our inflected word type, similar to our analysis of the accuracy data. Similarly, we ran 2 Morphological Complexity × 2 Task Type repeated measures ANOVAs for the three derived word types. We wanted to see if the matched control words were read faster than the morphologically complex target words, regardless of which response type measure was used. In addition, we wanted to examine the interaction between morphological complexity and task type to see if the magnitude of difference in response times was smaller in the passage than in the naming task. If this turned out to be the case, it would indicate that contextual clues played an important role.

It is important to note that for the main effect of task type, we could not directly compare differences between the naming task and the passage because response time data were collected differently. Instead, we expected that the response times would always be faster in the passage than in the naming task for two reasons: the context was useful in the accuracy data, and the response type measure in the passage did not measure the time it took participants to process the word. Results indicated that this was the case for all word types.

#### Inflected versus inflected matched control words

**Figure 2.**
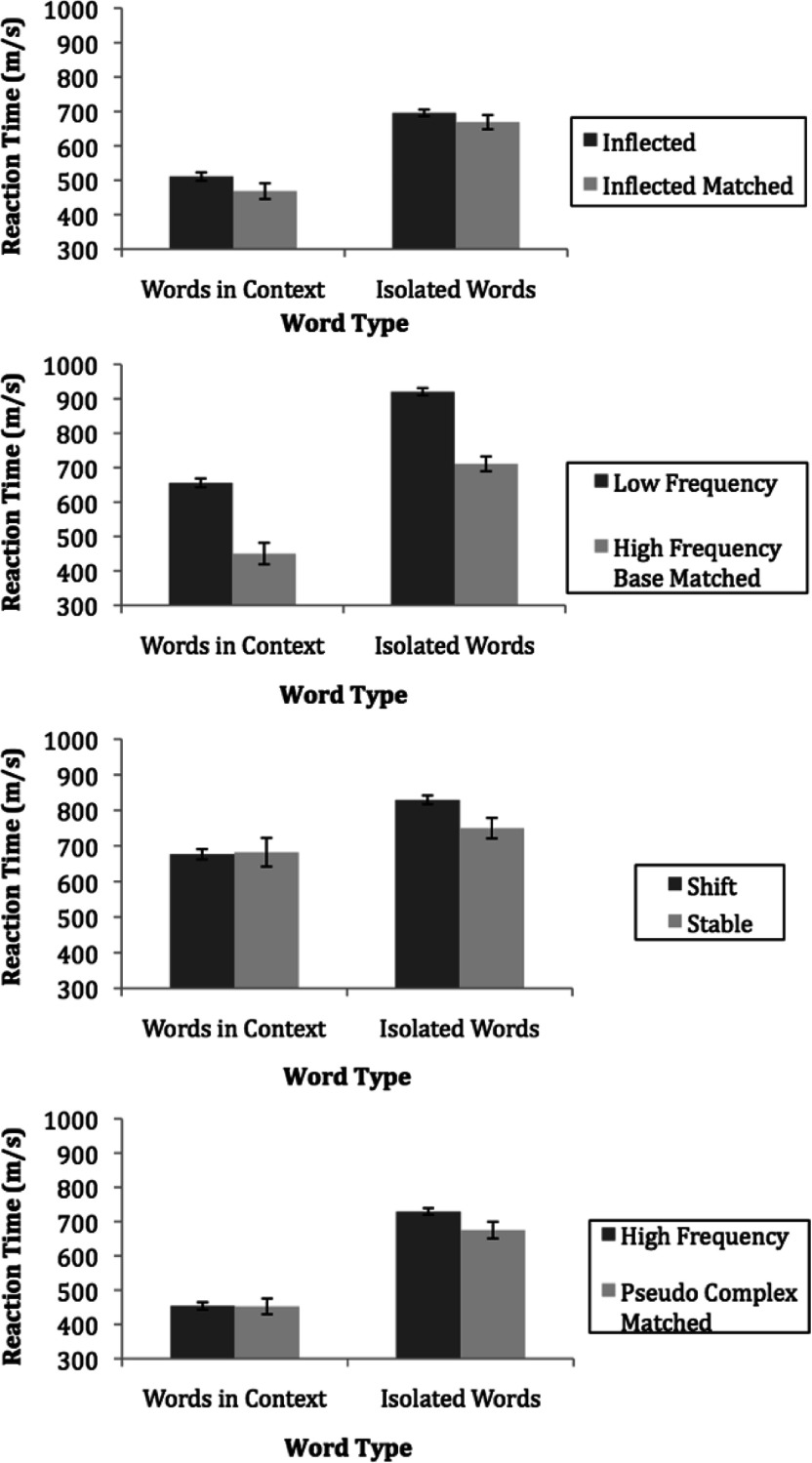
The mean response times on all word types in context and in isolation.

We found a significant main effect of morphological complexity, such that participants were significantly faster at reading the inflected matched control words (*M =* 568.43 ms) as compared with the inflected complex words (*M* = 603.33 ms), *F* (1, 47) = 20.41, *p* < .001. As expected, this finding demonstrates that adults with low literacy skills are vulnerable to morphologically complex words and have difficulty with them. The interaction between morphological complexity and task type was not significant, *F* (1, 47) = 1.21, *p* > .05 (see Figure 2, panel 1). Thus, the magnitude of difference in response times between inflected complex words and single-morpheme matched controls was no different in isolation as compared with the magnitude of difference in response times between inflected complex words and single-morpheme matched controls in context.

#### Low-frequency, high-frequency base versus high-frequency, high-frequency base words

There was a significant main effect of morphological complexity, such that the high-frequency base matched control words (*M* = 580.66 ms) were read faster than low-frequency, high-frequency base words (*M* = 787.97 ms), *F* (1, 46) = 276.34, *p* < .001. There was not a significant interaction between morphological complexity and task type for low-frequency versus high-frequency base matched control words, *F* (1, 46) = 0.018, *p* > .05 (see Figure 2, panel 2). Therefore, the magnitude of difference in response times between the low-frequency words and the high-frequency base matched control words in the isolated word naming task was approximately the same as the magnitude of difference in response times between the low-frequency and the high-frequency base matched control words found in context.

#### Phonological shift versus stable words

There was not a main effect of morphological complexity, *F* (1, 30) = 3.25, *p* = .082, but there was a significant interaction between morphological complexity and task type for phonological shift versus stable matched control words, *F* (1, 30) = 4.82, *p* = .036. Interaction contrasts demonstrated that the magnitude of difference in response times between the shift words (829.51 ms) and the stable matched control words (749.97 ms) was significantly different in the naming task, but the difference in response times between the shift (676.68 ms) and the stable words (682.30 ms) was eliminated when the words were encountered in context, *F* (1, 30), = 4.82, *p* = .036 (see Figure 2, panel 3).

#### Complex versus pseudocomplex words

There was a significant main effect of morphological complexity, such that the pseudocomplex matched control words (*M* = 563.66 ms) were read faster than the complex words (*M* = 591.54 ms), *F* (1, 49) = 10.49, *p* = .002. In addition, there was a significant interaction between morphological complexity and task type for complex versus pseudocomplex matched control words, *F* (1, 49) = 10.15, *p* = .003. Interaction contrasts revealed that the magnitude of difference in response times between complex words (*M* = 729.20 ms) and pseudocomplex words (*M* = 674.89 ms) was significantly different in the naming task, but the difference in response times between complex (453.89 ms) and pseudocomplex words (452.42 ms) was eliminated in context, *F* (1, 49), = 10.15, *p* = .003 (see Figure 2, panel 4).

## DISCUSSION

The purpose of this study was to examine morphological skills in a sample of adults with low literacy. First, we wanted to assess the contribution of morphological awareness, phonological awareness, and decoding to reading comprehension. Second, we wanted to investigate the accuracy and response times on different types of printed morphologically complex words as compared to matched control words in an oral reading passage and a word naming task. Results indicated that the majority of our hypotheses were supported. Both morphological and phonological awareness were found to be unique predictors of reading comprehension. Further, morphological awareness contributed additional variance beyond phonological awareness. For morphological processing, adults were faster and more accurate at reading matched control words than morphologically complex words in both the passage and the naming task. Moreover, words presented in context were read faster and more accurately than words presented in isolation. Similar to research that has been done with children, these findings suggest that morphological awareness is an important contributor to reading comprehension in adults with low literacy. The findings also indicate that adults with low literacy skills are vulnerable to the morphological complexities of printed words. This study is the first to examine morphological awareness and morphological processing in adults with low literacy and has important implications for understanding adults’ acquisition of literacy skills and for instructional practices in ABE programs.

### Predictors of reading comprehension

Morphological awareness has been found to play a significant role in reading comprehension, after controlling for phonological awareness and word decoding skills in children (Deacon & Kirby, 2004; Kirby et al., 2012). Although there have been no studies with ABE students looking at morphological awareness as a predictor of reading comprehension, we hypothesized that similar to studies of children, morphological, phonological, and decoding skills as a set would predict adults’ reading comprehension. Results revealed that phonological and morphological awareness were unique predictors of reading comprehension. Further, these predictors accounted for a large proportion of the variance in reading comprehension, suggesting the importance of morphological and phonological skills to reading comprehension in this population. Although decoding did not emerge as a significant unique predictor of reading comprehension, we do not take this to mean that decoding is not an important contributor to adults’ reading comprehension. Decoding was moderately to highly correlated (*r* = .61) with reading comprehension. Instead, we believe that there is a high degree of multicollinearity among phonological, morphological, and decoding skills. Therefore, decoding skills are unable to account for additional variance in reading comprehension beyond phonological and morphological skills.

Previous literature on phonology and morphology has contested the degree to which phonological awareness and morphological awareness are separate, individual predictors of reading skills. Until recently, phonological awareness was thought of as the single most important predictor of reading achievement in children (Carlisle, 2003). Consistent with research with children, we predicted that after controlling for phonological awareness, morphological awareness would be a unique predictor of adults’ reading comprehension skills. We found that morphological awareness accounted for additional variance beyond phonological awareness. This is an important finding because it establishes that morphological awareness is an important contributor to the reading comprehension skills of adults enrolled in ABE programs. The educational implications of this finding are discussed in a section on implications below.

### Accuracy and response times on inflected words

Because we found morphological awareness to be an important contributor to reading comprehension, we wanted to investigate different characteristics of printed morphologically complex words to which adults may be sensitive. We investigated accuracy and response times on inflected morphologically complex words compared to single-morpheme matched control words. Few studies with older children have incorporated words with inflected endings because knowledge of inflectional morphology is mastered by the early elementary school years (Anglin, 1993; Berko, 1958; Wysocki & Jenkins, 1987). Worthy and Viise (1996) compared adults in literacy classes with children matched on achievement level for spelling and reported that adults had particular difficulties with inflectional endings. Thus, we expected slight deviations from the children's research: we proposed that inflectional morphology presents more of a challenge for the population of adults with low literacy skills. Our results demonstrated this trend: adults were less accurate and slower on inflected complex words compared to single-morpheme matched control words in reading a passage and in a naming task. Therefore, adults with low literacy struggle with inflected complex words in print. Incorporating explicit teaching of inflectional endings in ABE programs is imperative to help adults parse complex words.

### Accuracy and response times on derived words

Next, we looked at the morphological characteristics of word frequency and word transparency in derived morphologically complex words compared to matched control words. We hypothesized that participants would demonstrate greater accuracy and respond faster to all three of the derived matched control word types as compared to the three types of derived complex word types in both the oral reading passage and the naming task. Results indicated that for all derived word types, adults with low literacy skills were more accurate on matched control words than on complex words. In addition, participants were faster at responding to matched control words than to complex words for two of the word types: high-frequency, high-frequency base words and pseudocomplex words. Response times on the phonological transparency word type were the only ones not supported by our hypothesis: stable matched control words were read as quickly as shift words.

In general, these findings are consistent with past literature, which reports that frequency, familiarity, and transparency of words play an important role in determining a reader's awareness of morphological structure. Carlisle and Stone (2005) found that derived words that were phonologically transparent and high frequency were read more accurately and faster than phonological shift and low-frequency words for children. These findings are consistent with the present study for adults: stable and high-frequency words were processed more accurately than were phonological shift and low-frequency words.

Our finding that there were no differences in response times between phonological shift and stable words deviated from Carlisle and Stone's (2005) results. Looking at this finding, we noticed that there was a large discrepancy in response times within the naming task such that shift words were responded to at a much slower rate than were stable words. There was no difference in response times for shift and stable words within the oral reading passage even though participants were more accurate on stable words in both the passage and the naming task. We think that this difference in response times is eliminated in the passage because adults utilized contextual clues to help them read words faster. The shift and stable words were the most difficult words within the tasks; therefore, many of the adults who struggled with these words were eliminated from the response time data because they did not produce any correct words. Thus, adults who actually were able to read some of these words probably performed better on the task and utilized contextual clues to help them identify the words faster. In isolation, the adults did not have the clues to help them figure out the more difficult phonological shift words, and they therefore spent longer trying to identify the words.

Carlisle and Stone (2005) also found that high-frequency words were read more accurately than pseudocomplex words. This study did not replicate that finding: the adults read the pseudocomplex words both more accurately and faster than the high-frequency complex words. In contrast to the Carlisle and Stone (2005) study, we hypothesized that the pseudocomplex words would be read more accurately and faster because we proposed that adults possess the ability to engage in morphological decomposition as opposed to processing complex words holistically. Thus, adults with low literacy skills recognized the base in the high-frequency complex word forms but had less developed morphological awareness to efficiently process and accurately identify the derived form. Participants did not need to break down the single-morpheme pseudocomplex words and instead read these words holistically. Both the high-frequency complex and pseudocomplex were high-frequency words; we surmise that adults struggled to decompose and produce the derived complex form.

### Importance of context

This study expanded on the Carlisle and Stone (2005) study by examining accuracy of word reading by looking at words presented in context versus words presented in isolation. The exposure to the interactive nature of spellings, pronunciations, syntax, and meanings present in a passage could influence accuracy and speed of word recognition. Adults have more exposure to language and printed materials than children. Adults with low literacy skills are no exception; this group may know a spoken word but may not be able to identify the written form of that word. We expected that context would be a positive factor for word identification because context provides clues to infer meaning and help recognize unfamiliar words. Results supported this hypothesis: adults were more accurate at identifying morphologically complex words in context for three of our word types. This finding was especially salient for phonological shift words: for words presented in isolation, only 34 participants got at least one word correct and averaged a 36.5% word accuracy level. Conversely, for words presented in context, all 55 participants were included and averaged a 79.2% word accuracy level.

The only word type that did not follow this trend was the high-frequency complex words versus pseudocomplex matched control words. We propose that the words were too familiar to the adults because complex and pseudocomplex words were high frequency and relatively easier words in relation to the other derived word types in this study. Thus, the presentation of the words (in context vs. in isolation) was irrelevant because overall the participants had high accuracy levels on both complex and pseudocomplex words.

### Limitations

There are a few limitations of this study that should be addressed. First, the diversity and the size of the sample posed a limitation: the 57 participants spanned an array of ages, language experience, and class levels. The study included both ESOLs and native English speakers. Further, the study did not yield enough power to be able to divide the participants by class: only 11 ESOL students, 12 pre-GED students, and 29 GED students completed all tasks. While it would have been beneficial to examine differences across groups, it is important to note that our sample is representative of the population of adults enrolled in ABE programs across the United States (National Research Council, 2012). In addition, in a recent study, Binder et al. (2011) used a similar population of adults with low literacy skills and found no differences between the native and nonnative reader groups in how different component skills (e.g., phonological and orthographic awareness and oral reading fluency) predicted reading ability. However, we recognize that this continues to be an issue that warrants further investigation. Second, there were difficulties with the naming task. Many of the word types included fewer words than the oral reading passage. By including fewer words in the naming task, there was little room for errors: if all words were missed, an entire participant was eliminated from the analyses. Third, response time data were measured differently between the passage and the naming task, and therefore it was difficult to interpret the findings. It would be beneficial and more useful to directly compare response times between the two task types to see if accuracy data corresponded with response time data.

### Future research

There are several important directions to consider for future research on morphological awareness and processing and reading abilities in adults with low literacy skills. It would be interesting to differentiate by ability levels and investigate morphological skills from adults with low literacy skills in beginning literacy programs through those in GED-level classes. Comparing adults with low literacy skills to children based on achievement level could allow one to assess differences in the developmental trajectory of acquiring morphological rules. Our study found that adults with low literacy skills struggled with both inflected and derived complex words. Few studies have included inflected and derived words and none assessing both children and adults with low literacy. This type of study could have important implications for the most effective approaches to teaching adults about morphological rules. Finally, because past research has investigated the relationship among phonology, morphology, and orthography in children, it would be worthwhile assessing these relationships in adults by including orthographic knowledge.

### Educational implications

The results of the current study indicate that morphological awareness is an important predictor of adults’ reading comprehension over and above phonological awareness. In addition, the study indicates that adults are vulnerable to morphological complexity. This study enhances the existing literature on adults with low literacy and demonstrates that morphological awareness is an important component skill of reading comprehension for this population. This could have important implications for ABE programs because explicitly teaching adults to understand morphological rules and how to decompose words into constituent morphemes could improve their morphological awareness, vocabulary knowledge, and subsequently reading comprehension skills. For children, understanding morphemic structure aids them in understanding and figuring out unfamiliar words and directly corresponds to spelling. The importance of rules regarding morphology and spelling contradicts some of the phoneme–grapheme correspondences when alternative pronunciations exist for word endings that are spelled the same way. For example, the past tense inflection, *–ed*, presents a challenge because *kissed* (*/t/* sound), *killed* (*/d/* sound), and *waited* (/-*əd/* sound) are all spelled the same but pronounced differently (Carlisle, 2010). Nunes and Bryant (2009) proposed a reading program emphasizing spelling abilities by directly teaching morphemic spelling rules. This type of instruction would allow learners to focus more on word structure (learning prefixes and suffixes) and enable them to apply these rules to promote reading comprehension and expand vocabulary knowledge. Explicit morphological instruction (in both inflectional and derivational morphology) may be beneficial for adults with low literacy skills because they struggle with morphologically complex words. Spelling research has found that this group suffers from poor understanding of morphological structure and underdeveloped phonological skills; however, they have better orthographic knowledge in comparison with children matched on achievement level (Viise, 1996; Worthy & Viise, 1996). Our study finds that adults also struggle with morphologically complex words in passage reading and when identifying words presented in isolation. Therefore, an integrative instructional approach encompassing phonological, morphological, and orthographic knowledge should be explored in adult literacy programs.

## NOTE

1. Note that we reran all of our analyses excluding the 12 ESOL participants specifically from the ESOL class. Although this reduces power (*n* = 45), we were still able to replicate all of the same results for both accuracy and response times with these participants omitted.

## APPENDIX A

### Oral reading passage

**Bold** words are from the Carlisle and Stone (2005) study; *italicized* words are inflected types.

Long before **colonial** *times*, in the **hilly** *region* of France, there was a **silly** young prince in *search* of a **lover**. All his wealth did not make a **difference** because he was very lonely in his *large*, **empty** *palace*. He *wanted* a **lady** to cook him **dinner** and clean his **dirty** home. He *needed* to add **flavor** to his **cookery** and style to his dull *house.* But first he had to make a **confession**—before he was a prince, he was a **beggar** and a *thief.* He *entered* the lottery and became a **winner.** Despite his financial **security**, he was afraid and **secretive.** It was a **fearsome** worry that someone would discover his *past*.

One day a **pretty** woman *wearing* a **stylish** dress with a **flowery** design *walked* by the castle. She was *singing* a beautiful melody. He *looked* at her with **intensity** and *liked* her **sparkly** *earrings* and *blonde* hair. He was drawn to her **natural** *beauty*. He went up to her with **confidence** and invited her to the annual music **convention.** *Realizing* his **sincerity** and *charming* good *looks*, she said yes. They sat on the *grass beneath* a **shady** *tree* to enjoy the **serenity** of the afternoon and *discuss* their *mutual* **preference** for **classical** music. He took her to the *opera* and the *ballet.*

The prince could not stop *smiling* because he felt so **lucky.** The couple began *spending* every moment together. The prince's *friends* took his **dependence** on her for **stupidity** and lack of **maturity.** He was hurt by the **severity** of their opinions. But he did **hover** and cling to her the **majority** of the time. He gave her a *hundred presents* and *asked* her to *marry* him. She *explained* her **cultural** *beliefs*—*since* she was the youngest daughter, she had to *stay* home and take care of her mother. He looked at her in **puzzlement** and his heart broke to *pieces*. He was alone again, still in search of his princess.
